# Human papillomavirus prevalence and genotypes in Gulf Cooperation Council countries: A scoping review 2017-2024

**DOI:** 10.5339/qmj.2024.33

**Published:** 2024-07-31

**Authors:** Nahlah AlMesbah, Jihene Maatoug, Nagah Selim, Iheb Bougmiza

**Affiliations:** 1 Community Medicine Residency Program, Medical Education, Hamad Medical Corporation, Doha, Qatar *Email: almesbahnahlah@gmail.com; 2 Community Medicine Residency Program, Primary Health Care Corporation, Doha, Qatar; 3 Faculty of Medicine, Sousse University, Tunisia; 4 Public Health and Preventive Medicine, Faculty of Medicine, Cairo University, Egypt; 5 College of Medicine, QU Health, Qatar University, Doha 2713, Qatar

**Keywords:** Human papillomavirus, genotype, prevalence, Qatar, Kuwait, Saudi Arabia, Oman, Bahrain, United Arab Emirates

## Abstract

**Background:**

Cervical cancer remains a global health challenge, claiming the lives of millions annually and having a significant impact on Gulf Cooperation Council (GCC) countries. Human papillomavirus (HPV), the primary causative agent, plays a central role, with regional variations in prevalence.^1^ The process from HPV infection to neoplastic changes takes 5–25 years to occur, hence, knowing its prevalence in our community is vital.^2^

**Methods:**

PubMed and SCOPUS were searched to identify articles related to cervical and anogenital HPV prevalence and genotypes in Qatar, Kuwait, Bahrain, Oman, the United Arab Emirates (UAE), and the Kingdom of Saudi Arabia (KSA) published between 2017 and 2024.

**Results:**

A total of 19 articles were included in this review. Eight studies were from KSA, four were from Kuwait, three were from the UAE, one was from Qatar, Oman, and Bahrain, and one presented data collectively from the KSA, UAE, Qatar, and Bahrain. The prevalence of HPV ranged between 4.7% and 77% in studies from the KSA, between 15% and 54.3% in studies from Kuwait, between 14.7% and 88% in studies from the UAE, was 8.1% and 31.3% in the two studies from Qatar, and was 17.8% and 20% in the studies from Oman and Bahrain, respectively. HPV 16 was the most prevalent high-risk genotype found in studies conducted in the KSA, UAE, Kuwait, and Qatar. In Oman, HPV 82 predominated. In Bahrain, the majority had other non-HPV 16/18/45 genotypes. In the UAE and Kuwait, HPV 11 was the predominant low-risk type, followed by HPV 6. In Qatar, HPV 81 was the most common low-risk type, followed by HPV 11. In Oman, HPV 54 was the most common low-risk type, followed by HPV 42.

**Conclusion:**

There are no studies with data on HPV prevalence and genotypes among women who have been vaccinated against HPV in GCC countries.

## 1. Introduction

**Figure 1.**
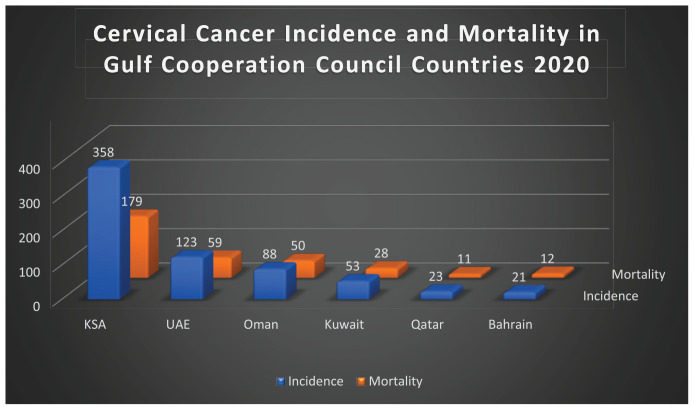
Bar chart illustrating cervical cancer incidence and mortality in GCC countries (data from 2020).

Cervical cancer is the fourth most common cancer among women globally, following breast, colorectal, and lung cancer.^3,4^ In 2020, there were 604,000 new cases of cervical cancer and 342,000 deaths reported worldwide.^4^ Approximately 90% of these deaths occurred in low- and middle-income countries, which is mainly due to the inequitable access to human papillomavirus (HPV) vaccination, screening services, and treatment for women diagnosed with cervical cancer.^4^ Estimates from 2020 show that the incidence of cervical cancer in Gulf Cooperation Council (GCC) countries is highest in the Kingdom of Saudi Arabia (KSA), followed by the United Arab Emirates (UAE), Oman, Kuwait, Qatar, and finally Bahrain.^5-10^ Mortality from cervical cancer in GCC countries follows the same order except that Qatar had a lower mortality than Bahrain (details are presented in Figure 1).^5-10^

HPV is the primary factor responsible for cervical cancer. Evidence from virological, molecular, clinical, and epidemiological investigations has demonstrated that cervical cancer results from persistent, unresolved infection with certain HPV genotypes.^1^ It has been estimated that 99% of preinvasive and invasive cervical lesions are associated with HPV.^2,11^ Similarly, HPV has been implicated in causing 65% of vaginal cancers, 50% of vulvar cancers, and 45%–90% of oropharyngeal cancers.^2^ Furthermore, a notable association between HPV and anal cancer is evident, with at least 80% of anal cancer patients testing positive for HPV.^12^ Additionally, a systematic review found that 47.9% of penile cancer cases tested positive for HPV.^13^ Infection with HPV is often asymptomatic and resolves spontaneously.^14,15^ However, infection with low-risk HPV 6 and 11 often manifests as condyloma acuminatum (anogenital warts).^14,16,17^

Two hundred HPV genotypes have been documented; however, only 12 have been designated as carcinogenic. HPV 16 contributes to 50% of cervical cancer cases, while HPV 18 is responsible for 10%. Furthermore, HPV 16 and 18 increase cancer risk by 435 and 248 times, respectively. HPV 16 and 18 have been detected in 99.7% of cancer cases globally. HPV 31, 33, and 45 have been linked to 5% of cases; HPV 52 has been linked to 3% of cases; and HPV 35 and 58 have been linked to 2% of cases each.^16^

It takes approximately 6–24 months for HPV to be cleared by the body. Cervical HPV clearance takes approximately 9.6 months, whereas genital HPV clearance takes approximately 7.6 months.^14^ The process from HPV infection to neoplastic changes takes 5 to 25 years to occur.^2^

This review aims to identify HPV prevalence and the common genotypes in GCC countries, as well as gaps that should be addressed in future research.

## 2. Methods

### 2.1. Review approach

PubMed and SCOPUS were searched until March 30, 2024, to identify articles related to cervical and genital HPV prevalence and genotypes in the six GCC countries: Qatar, Kuwait, Bahrain, Oman, the UAE, and KSA. The following MeSH terms were used: “human papillomavirus,” “HPV,” “genotype,” “Kuwait,” “Qatar,” “United Arab Emirates,” “Kingdom of Saudi Arabia,” “Oman,” and “Bahrain.” Specific details of the search strategy can be found in Table 1.

### 2.2. Inclusion and exclusion criteria

Original articles about cervical and anogenital HPV prevalence and genotypes with data from GCC countries that were published between 2017 and 2024 were included. There were no exclusion criteria.

### 2.3. Article screening and selection

**Figure 2.**
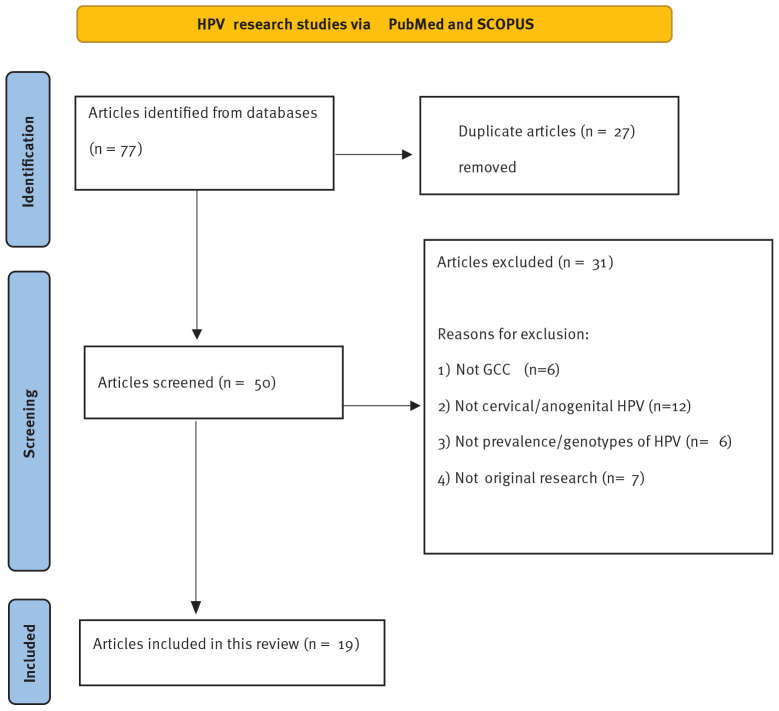
Flow diagram illustrating the process of article inclusion.

The initial search generated a total of 77 articles. After removing 27 duplicate articles, 50 remained and were screened. A total of 31 articles were excluded, six of which were not from GCC countries, 12 of which were not about cervical or anogenital HPV, six of which were not about the prevalence or genotypes of HPV, and seven of which were not original research papers. Figure 2 demonstrates the article screening process.

### 2.4. Data extraction and analysis

All articles were reviewed, and the following variables were extracted into a Microsoft Word document: author, year published, timeline of data collected, sample size, study design, country, and findings. This data was then summarized as frequencies.

## 3. Results

This review included a total of 19 articles. Of these, eight originated from the KSA, four from Kuwait, and three from the UAE. Additionally, one study each was from Qatar, Oman, and Bahrain. One study presented combined data from the KSA, UAE, Qatar, and Bahrain.

During the period from 2017 to 2024, the publication frequency of studies varied each year. Two studies were published in both 2017 and 2018. The highest publication frequency occurred in 2020, with four studies. In the years 2019, 2021, and 2023, three studies were published each year. Notably, only one study was published in both 2022 and 2024.

All studies were cross-sectional, except one, which was a case series.^18^ The sample size ranged between 75 and 2,478.

All studies were conducted among females except one, which was conducted among men and women with anogenital warts.

The study period covered in the studies ranged from 6 months up to 22 years.

### 3.1. Prevalence of HPV in UAE

The prevalence of HPV was 14.7% and 60.57% in the studies by Ali et al.^19^ and Odeh et al.^20^ respectively. In the study by Odeh et al.^20^ 28.6% of the HPV-positive samples were found to have abnormal cytology. The prevalence of high-risk HPV was 56.1% and 88% among women with abnormal cytology in the studies by Revannasiddappa et al.^21^ and Albawardi et al.^22^ respectively.

### 3.2. Prevalence of HPV in KSA

The prevalence of HPV was 4.7%, 5.9%, 14.5%, 17.2%, 18%, and 30.4% in the studies by AlShammari et al.^23^ Mousa et al.^24^ Kussaibi et al.^25^ Ali et al.^19^ Obeid et al.^26^ and Alhamlan et al.^27^ respectively. The prevalence of HPV was 52.9% among women with abnormal cytology in the study by Faqih et al.^18^ and 77% among cervical cancer samples in the study by Alsbeih et al.^28^

### 3.3. Prevalence of HPV in Kuwait

The prevalence of high-risk HPV was 1.7% among cases with normal cytology, 19% among cases with atypical squamous cells of undetermined significance (ASCUS), and 35.3% among cases with endocervical abnormalities in the study by Kapila et al.^29^ The prevalence of common wart viruses was the highest (50.6%) among men and women with anogenital warts in the study by Al-Awadhi et al.^30^ This study also found that the prevalence of high-risk HPV was 34.62% and the prevalence of low-risk HPV was 14.1% among the same population. The prevalence of high-risk HPV was 30.57% among women with abnormal cytology in the study by Mallik et al.^31^ The prevalence of high-risk HPV was 61%, low-risk HPV was 36%, and intermediate-risk HPV was 3% among samples with abnormal cytology in the study by AlRoomy et al.^32^

### 3.4. Prevalence of HPV in Qatar

The prevalence of HPV was 8.1% in the study by Elmi et al.^33^ The prevalence was higher among Qatari women (9.8%), compared to non-Qatari Arab women (6.1%). It was also higher among women with abnormal cytology (16.7%), compared to women with normal cytology (7.6%).

The HPV prevalence was higher at 31.3% in the study by Ali et al.^19^

### 3.5. Prevalence of HPV in Oman

The prevalence of HPV was 17.8% in the study by Al-Lawati et al.^34^ HPV prevalence was also higher among women with abnormal cytology (37.5%) compared to women with normal cytology (16.9%).

### 3.6. Prevalence of HPV in Bahrain

The prevalence of HPV was 20% in the study by Ali et al.^19^

### 3.7. Common genotypes in UAE

The study by Odeh et al.^20^ concluded that high-risk HPV 16 (9.61%) and HPV 45 (7.69%) were the most common. As for the low-risk genotypes, HPV 6 (13.46%), HPV 11 (9.61%), and HPV 62/81 (7.69%) were the most common among 104 women.

The most common high-risk HPV genotypes among 422 women with cervical abnormalities in the study by Revannasiddappa et al.^21^ were HPV 16 (15.2%), followed by HPV 31 (11.7%), HPV 53 (8.9%), HPV 66 (8.6%), and HPV 51 (8.3%).

In the study by Albawardi et al.^22^ which was conducted among 75 women with a diagnosis of high-grade squamous intraepithelial lesion (HSIL) or cervical cancer, the most common genotypes were HPV 16 (49%), HPV 31 (20%), and HPV 18 (6.6%).

*In summary, HPV 16 was the most common high-risk HPV type in three studies from the UAE*.^20-22^ *HPV 31 was the second most common high-risk type in two studies*.^21,22^ *HPV 58 was the second most common high-risk type in one study*.^20^ *HPV 11 was the most common low-risk type, followed by HPV 6 in one study*.^20^ Table 2 outlines further details from all the studies included in this review.

### 3.8. Common genotypes in KSA

The common genotypes among 14 HPV-positive women were HPV 16 (42.9%), HPV 52 (21.4%), HPV 58 (14.3%), and HPV 33 (14.3%) in the study by AlShammari et al.^23^

The genotypes detected in the study by Alhamlan et al.^27^ among 96 HPV-positive women were HPV 16 (56.3%), HPV 18 (7.3%), HPV 31 (4.2%), HPV 58 (3.1%), HPV 33 (2.1%), HPV 56 (2.1%), HPV 35 (1%), HPV 45 (1%), and HPV 82 (1%). The remaining samples (19%) had multiple infections involving HPV 6, 11, 51, 53, 56, 57, 66, 68, and 71.

Kussaibi et al.^25^ found that among 24 HPV-positive women, the majority (33.3%) tested positive for HPV 16. Additionally, 8.3% tested positive for HPV 16 and HPV 18/45 coinfections. The remaining 58.3% had other high-risk HPV types that were not mentioned.

In the study by Mousa et al.^24^ out of 119 vaginal swabs, 5.9% tested positive for HPV 10, 11, 58, 62, 66, and 67.

In the study by Alsbeih et al.^28^ out of 163 HPV-positive cervical cancer samples, HPV 16 (75%) and 18 (9%) were the most common. Among 90% who had a single infection, HPV 16 predominated (67.5%) and was followed by HPV 31 (6.8%), HPV 18 and 45 (1.8% each), HPV 73 (1.8%), HPV 59 (1.3%), and HPV 6, 56, and 64 (0.6% each). The remaining 10% had double infections involving HPV 33, 35, 39, 51, 52, 70, and 82.

Faqih et al.^18^ found that among 82 HPV-positive women, 58.6% tested positive for a single infection with HPV 31, 33, 35, 39, 51, 52, 56, 58, 59, 66, and 68. The prevalence of coinfection with the previously mentioned HPV types in combination with HPV 18/45 was 6.1%. The prevalence of HPV 16 was 22%, and that of HPV 18/45 was 7.3%.

In the study by AlOtaibi et al.^35^ out of 142 HPV-positive samples, 49.2% tested positive for HPV 16, 25.3% tested positive for HPV 18 and 7% tested positive for HPV 31.

In the study by Obeid et al.^26^ out of 121 HPV-positive samples, a single infection with HPV 16 was the most common (51%), followed by HPV 18 (28%), HPV 56 and 58 (3.3% each), HPV 31 and 42 (1.7% each), and HPV 33, 51, 6, 66, and 61 (0.8%. each). Multiple HPV types were positive in 7% of the samples.

*In summary, HPV 16 was the most common high-risk HPV type in six studies*.^23,25-28,35^ *HPV 18 was the second most common high-risk type in four studies*.^26-28,35^ *HPV 52 was the second most common high-risk type in one study*.^23^ *The study by Faqih et al*.^18^ *was not included in the ranking of the genotypes as the results are presented for HPV 16, 18, and 45 only. The remaining genotypes were collectively grouped under the category of “other” HPV genotypes. The study by Mousa et al*.^24^ *was also not considered in the ranking of the genotypes, as the number of cases for each genotype was not mentioned.* Table 2 outlines further details from the studies.

### 3.9. Common genotypes in Kuwait

In a study by Kapila et al.^29^ out of 27 HPV-positive samples (the majority had ASCUS and endocervical abnormalities), 18.5% tested positive for HPV 16, 3.7% tested positive for HPV 18/45 and 29.6% tested positive for coinfection with HPV 16 and HPV 18/45. HPV 16 and HPV 18/45 were negative in 48.1% of the HPV-positive samples, indicating the presence of other high-risk HPV genotypes.

In the study by Al-Awadhi et al.^30^ which was conducted among 156 men and women with anogenital warts, 34.62% tested positive for high-risk HPV (HPV 16, 18, 33, and 38), 14.1% tested positive for low-risk HPV (HPV 6 and 81) and 50.6% tested positive for common wart viruses (HPV 1a, 2, 7, 27b, 27, 57b, 57c, and 65).

The study by Mallik et al.^31^ was conducted among cases with epithelial abnormalities. Out of 116 HPV positive cases that were genotyped, 62.93% tested negative for HPV 16, 18, and 45, whereas 31.04% tested positive for HPV 16, and 6.03% tested positive for HPV 18/45.

In the study by AlRoomy et al.^32^ out of 115 samples with high-risk genotypes among women with abnormal cytology, HPV 16 was the most prevalent (34%), followed by HPV 66 (13%). Additionally, out of 69 samples with low-risk genotypes, HPV 11 (36.2%) and HPV 6 (30.4%) were the most prevalent.

*HPV 16 was the most common high-risk HPV type in two studies*.^30,32^ *HPV 66 was the second most common high-risk type in one study*.^32^ *HPV 11 was the most common low-risk type, followed by HPV 6 in one study*.^32^ *The studies by Kapila et al*.^29^ *and Mallik et al*.^31^ *were not included in the ranking of the genotypes, as the results are presented for HPV 16, 18, and 45 only. The remaining genotypes were collectively grouped under the category of “other” HPV genotypes. Further details from the studies can be found in Table 2.*

### 3.10. Common genotypes in Qatar

In the study by Elmi et al.^33^ HPV 35 was the most common high-risk genotype among women with normal cytology (6.9%), whereas HPV 16 and 59 were the most common high-risk genotypes among women with abnormal cytology (25% each). The most common low-risk genotypes among women with normal cytology were HPV 81 (34.5%) and HPV 11 (31%), whereas the most common low-risk genotypes among women with abnormal cytology were HPV 11, 81, and 90 (25% each). Further details from this study and the study by Ali et al.^19^ which combined data from four countries, including Qatar, can be found in Table 2.

### 3.11. Common genotypes in Oman

In the study by Al-Lawati et al.^34^ the most common high-risk genotypes were HPV 82 (10.77%) and HPV 68 (7.69%). The most common low-risk genotypes were HPV 54 (12.31%) and HPV 42 (7.69%). Further details from the study can be found in Table 2.

### 3.12. Common genotypes in Bahrain

In the study by Alnoaimi et al.^36^ the majority (62%) had other non-HPV 16/18/45 genotypes, 24% had HPV 16, 6% had HPV 18/45, and 8% had coinfections. Further details from this study can be found in Table 2.

## 4. Discussion

This review reaffirmed the need for GCC countries to continue raising awareness about HPV vaccination and cervical cancer screening, given the wide distribution of both vaccine-preventable and non-vaccine preventable HPV genotypes in those countries. The prevalence of HPV ranged between 14.7% and 88% in studies from the UAE, between 4.7% and 77% in the studies from the KSA, between 15% and 54.3% in the studies from Kuwait, was 8.1% and 31.3% in the two studies from Qatar, and was 17.8% and 20% in the studies from Oman and Bahrain, respectively. In the context of this review, the HPV prevalence of 40.6% reported in an Iraqi study by Pity et al.^37^ aligns with the ranges observed in most GCC countries. However, the national prevalence of 7.8% in the Tunisian study by Ardhaoui et al.^38^ does not.

The prevalence of high-risk HPV among HSIL and cervical cancer cases was 88% in the UAE study by Albawardi et al.^22^ This was higher than the prevalence of 56.1% among women with cervical abnormalities in the UAE study by Revennasiddappa et al.^21^ The prevalence among cervical cancer patients in the KSA study by Alsbeih et al.^28^ was 77%, which falls between the prevalence rates reported in the UAE studies. Regardless, the HPV prevalence among cervical cancer cases was even higher at 91.7% and 92% in the Jordanian study by Abu-Lubad et al.^39^ and the English study by Torjesen,^40^ respectively.

The Kuwaiti study by Al-Awadhi et al.^30^ stands out as the sole study conducted among anogenital warts patients. The study reported a prevalence of 34.62% for high-risk HPV, which mirrors the prevalence in a Chinese study (34.5%) by Zhu et al.^41^ conducted among a similar population. Al-Awadhi et al.^30^; however, reported a low prevalence of low-risk HPV (14.1%) compared to 45.4% in the study by Zhu et al.^41^

This review revealed that HPV 16 is the most prevalent high-risk genotype found in studies conducted in the KSA, UAE, Kuwait, and Qatar. This differed from the Omani study, which showed that HPV 82 was the most common high-risk genotype. The findings from the KSA, UAE, Kuwait, and Qatar align well with the findings from most other countries. A study from Iran by Karimi-Zarchi et al.^42^ also found that HPV 16 was the most common high-risk genotype among women with ASCUS. Abate et al.^43^ conducted a study on cervical tissue samples in Ethiopia and Sudan and found that HPV 16 was the most common in both countries. Comparably, the Tunisian study by Ardhaoui et al.^38^ found that HPV 31 was the most common. The second most common high-risk genotype also varied among the countries. HPV 31, 18, 66, and 68 were the second most common high-risk genotypes in studies from the UAE, KSA, Kuwait, and Oman, respectively. Whereas, in Iran, Sudan, and Tunisia, HPV 31, 52, and 16 were the second most common high-risk genotypes, respectively.^38,42,43^

The most common low-risk HPV genotypes varied among the countries in this review. In the UAE and Kuwait, HPV 11 was the predominant low-risk type, followed by HPV 6. In Qatar, HPV 81 was the most common low-risk type, followed by HPV 11. In Oman, however, HPV 54 was the most common low-risk type, followed by HPV 42. To some extent, the findings from the UAE, Kuwait, and Qatar align with the results of an Egyptian study by Ibrahim et al.^44^ which found that HPV 6 was the most common low-risk type, followed by HPV 11.

None of the studies included data on HPV prevalence among HPV-vaccinated individuals. This is possibly due to the lack of an HPV vaccination program in the public healthcare system in GCC countries in the past years, as evidenced by the low self-reported HPV vaccination rates of 16% and 4.7% in studies from Kuwait and KSA, respectively.^45,46^ Additionally, studies from Qatar and KSA have demonstrated that 60% and 72.6%, respectively, were unaware that HPV is a cause of cervical cancer, which could further contribute to the low vaccine uptake in GCC countries.^47,48^

### 4.1. Strengths and limitations

A strength of our review is that data extraction from all 19 studies was conducted by a single reviewer to maintain consistency. A limitation, however, is that only two databases were used to conduct the literature search; thus, it is possible that we may not have captured all potentially relevant studies available in other databases. Additionally, some studies, although published between 2017 and 2024, included data from an older time period.

### 4.2. State-of-the-art

The field of cervical precancer is witnessing rapid innovation. A pioneering study by Darwish et al.^49^ has introduced an innovative approach to the early detection of cervical precancer, utilizing a state-of-the-art vision transformer with shifted patch tokenization. This method has demonstrated remarkable accuracy and generalization capabilities, marking a substantial advancement over traditional techniques. Concurrently, Pal et al.^50^ have introduced a deep metric learning-based framework for cervical precancer detection, an approach that exhibits improved specificity over previously utilized algorithms. These contributions, among others, offer promising avenues for combating HPV-related health conditions.

## 5. Conclusion

Our review showed that there is no data on HPV prevalence and genotypes among women who have been vaccinated against HPV in GCC counties. This gap could be due to the lack of an HPV vaccination program in the preceding years. In the coming years, this situation is expected to change as countries like Qatar and Kuwait have recently introduced the HPV vaccine into their public healthcare systems. Successful uptake of the HPV vaccine could be ensured through prioritizing awareness campaigns and education initiatives, clear communication about the safety and effectiveness of HPV vaccines, and evidence-based recommendations, which can help build trust in vaccination programs. This step is anticipated to contribute to better data availability on HPV prevalence and genotypes among vaccinated individuals in the region.

## Annex

**Table of abbreviations**

**Table:** 

**Abbreviations**	**Term**
ASCUS	Atypical squamous cells of undetermined significance
GCC	Gulf Cooperation Council
HPV	Human papillomavirus
HSIL	High grade squamous intraepithelial lesion
KSA	Kingdom of Saudi Arabia
UAE	United Arab Emirates

## Conflict of Interest Statement

The authors declare that the research was conducted in the absence of any commercial or financial relationships that could be construed as a potential conflict of interest.

## Authors’ Contributions

NM—conception and design of the study, screening, data extraction, analysis and interpretation, and writing of the manuscript; JM—analysis and interpretation, revised the manuscript, and approved the manuscript; NS—analysis and interpretation, revised the manuscript, and approved the manuscript; IB—analysis and interpretation, revised the manuscript, and approved the manuscript.

## Figures and Tables

**Table 1.** Database, search strategy, filters used, and the number of results generated.

**Database**	**Search strategy**	**Filters**	**Results**
Scopus	“HPV” OR “human AND papillomavirus” AND “genotype” AND “Kuwait”	Year range: 2017 to 2024	3
	“HPV” OR “human AND papillomavirus” AND “genotype” AND “Qatar”		4
	“HPV” OR “human AND papillomavirus” AND “genotype” AND “Oman”		1
	“HPV” OR “human AND papillomavirus” AND “genotype” AND “Bahrain”		2
	“Human papillomavirus” OR “HPV” AND “genotypes” AND “Saudi”		14
	“Human papillomavirus” OR “HPV” AND “genotypes” AND “United Arab Emirates”		5
PubMed	“Human papillomavirus” OR “HPV” AND “genotypes” AND “Kuwait”	Full text; From 2017 to 2024	6
	“Human papillomavirus” OR “HPV” AND “genotypes” AND “Qatar”		10
	“Human papillomavirus” OR “HPV” AND “genotypes” AND “Oman”		1
	“Human papillomavirus” OR “HPV” AND “genotypes” AND “Bahrain”		1
	“Human papillomavirus” OR “HPV” AND “genotypes” AND “Saudi”		23
	“Human papillomavirus” OR “HPV” AND “genotypes” AND “United Arab Emirates”		7

**Table 2.** Summary of all the studies included in this review.

**Author**	**Timeline**	**Summary**
**UAE**		
Odeh et al.^20^	Sept 2021–Apr 2022	• **Population: **women aged 20–59 years
		• **Sample size: **104
		• 63/104 (60.58%) women were found to have 112 different HPV genotypes.
		• 18/63 (28.6%) who tested positive for HPV had abnormal cytology and 45/63 (71.4%) had normal results.
		• Among the identified genotypes, 63 were categorized as low-risk and 49 as high-risk.
		• 25/104 (24%) had a single genotype and 38/104 (36.54%) had multiple genotypes.
		• The prevalence of a single high-risk genotype was 13/104 (12.5%). HPV 16 and 58 were the most prevalent among the single high-risk group (2/13, 15.4% each), followed by HPV 59, 73, 18, 66, 51, 45, 31, 68, and 82 (1/13, 7.6% each).
		• The prevalence of a single low-risk genotype was 12/104 (11.54%). HPV 11 was the most common (5/12, 41.7%), followed by HPV 6 (4/12, 33.3%) and finally HPV 40, 70, and 84 (1/12, 8.3% each).
		• Multiple genotypes additionally included the following types: HPV 42, 43, 44, 55, 62, 67, 81, 52, 86, 33, 35, 39, and 54.
		• *The study concluded that HPV 6 (13.46%), HPV 11 (9.61%), HPV 16 (9.61%), HPV 62/81 (7.69%), and HPV 45 (7.69%) were the most common.*
Revannasiddappa et al.^21^	Jan 2018 –Sep 2019	• **Population: **Women with cervical abnormalities who underwent HPV genotyping.
		• **Sample size: **422
		• 248/442 (56.1%) tested positive for HPV.
		• The prevalence of high-risk HPV was 48%.
		• The most common high-risk HPV genotype was HPV 16 (15.2%), followed by HPV 31 (11.7%), 53 (8.9%), 66 (8.6%), and 51 (8.3%).
		• The prevalence of HPV 18 was 3.8%.
		• Other high risk HPV types identified include HPV 52, 35, 58, 56, 59, 18, 39, 45, 82, 33, 68, 70, 73, 26, 42, and 69.
Albawardi et al.^22^	2012–2016	• **Population: **Women diagnosed with HSIL and cervical carcinoma.
		• **Sample size: **75 (out of which, 70 were HSIL cases, 1 was an adenocarcinoma in-situ case and 4 were invasive squamous cell carcinoma cases).
		• 66/75 (88%) tested positive for high-risk HPV.
		• 8/75 (10.6%) had a double HPV infection and 4/75 (5.3%) had a triple HPV infection.
		• HPV 16 was the most common (37/66, 56%) followed by HPV 31 (15/66, 22.7%), HPV 18 (5/66, 7.6%), HPV 33, 45 and 52 (4/66, 6.1% each), HPV 58 (3/66, 4.5%), HPV 68, 35, 39, and 66 (2/66, 3% each), and HPV 59 and 51 (1/66, 1.5%).
		• The genotypes that were found as coinfections include HPV 16, 18, 31, and 33, among 5, 2, 4, and 1 case, respectively.
**KSA**		
AlShammari et al.^23^	May 2020–May 2021	• **Population: **women referred to do a Pap smear.
		• **Sample size: **300
		• 14/300 (4.7%) tested positive for HPV.
		• 8/14 (57%) of those who tested positive for HPV exhibited abnormal cytology, while 6/14 (42.9%) showed normal results.
		• The most common genotypes were: HPV 16 in 6/14 (42.9%), HPV 52 in 3/14 (21.4%), HPV 58 in 2/14 (14.3%), and HPV 33 in 2/14 (14.3%).
Alhamlan et al.^27^	2006–2016	• **Population: **Women aged 23–95 years.
		• **Sample size: **315
		• HPV was detected in 96/315 (30.4%) of patients.
		• 75/96 (78%) had high-risk HPV types detected.
		• 54/96 (56.3%) had HPV 16, 7/96 (7.3%) had HPV 18, 4/96 (4.2%) had HPV 31, 2/96 (2.1%) had HPV 33, 1/96 (1%) had HPV 35, 1/96 (1%) had HPV 45, 2/96 (2.1%) had HPV 56, 3/96 (3.1%) had HPV 58, and 1/96 (1%) had HPV 82.
		• 21/96 (21.9%) of the HPV positive samples had multiple infections, which included HPV 6, 11, 51, 53, 56, 57, 66, 68, and 71.
Kussaibi et al.^25^	2013–2019	• **Population: **Saudi women who had ASCUS on Pap smear testing and those at high risk of infection were investigated for HPV.
		• **Sample size: **164
		• 24/164 (14.5%) were positive for high-risk HPV.
		• 10/24 (41.7%) of those who tested positive for high-risk HPV had abnormal cytology.
		• HPV16 and HPV18/45 coinfection was detected in 2/24 women (8.3%).
		• HPV 16 was detected in 8/24 women (33.3%).
		• 14/24 (58.3%) had other high-risk HPV.
Mousa et al.^24^	Oct 2017–Apr 2018	• **Population: **women attending the gynecology clinic.
		• **Sample size: **119
		• 7/119 (5.9%) samples tested positive for HPV 10, 11, 58, 62, 66, and 67.
Alsbeih et al.^28^	1990–2012	• **Population: **Cervical cancer patients with histologically proven invasive tumors.
		• **Sample size: **232 total, but HPV testing was only done for 213.
		• 163/213 (77%) were HPV-positive.
		• 147/163 (90%) had a single HPV infection, with HPV 16 affecting the majority (110/163; 67.5%), followed by HPV 31 (11/110; 6.8%), HPV 18 and HPV 45 (9/163; 5.5% each), HPV 73 (3/163; 1.8%), HPV 59 (2/163; 1.3%), and HPV 6, HPV 56, and HPV 64 (1/163; 0.6% each).
		• The remaining 16/163 (10%) had double infections and involved additional HPV genotypes including: HPV 33, 35, 39, 51, 52, 70, and 82.
		• When both single and double infections are combined, HPV 16 remained the most common with an overall prevalence of 75%, followed by HPV18 (9%) and HPV 31 and HPV 45 (7% each).
Faqih et al.^18^	Jan 2021–Dec 2022	• **Population: **Women with abnormal cytology.
		• **Sample size: **155
		• 82/155 (52.9%) tested positive for HPV.
		• 48/82 (58.6%) tested positive for a single infection of HPV 31, 33, 35, 39, 51, 52, 56, 58, 59, 66, and 68.
		• 5/82 (6.1%) tested positive for the above HPV genotypes in combination with HPV 18/45.
		• 18/82 (22%) tested positive for HPV 16.
		• 6/82 (7.3%) tested positive for HPV 18/45.
AlOtaibi et al.^35^	N/A	• **Population: **HPV-positive cervical samples were obtained from previous studies.
		• **Sample size: **351 total samples, out of which 142 previously tested as HPV positive
		• 70/142 women (49.2%) tested positive for HPV 16.
		• 36/142 women (25.3%) tested positive for HPV 18.
		• 10/142 women (7.0%) tested positive for HPV 31.
		• The most common HPV genotypes were HPV 16 followed by HPV 18.
Obeid et al.^26^	N/A	• **Population: **women who underwent cervical screening.
		• **Sample size: **933
		• 165/933 (18%) specimens were positive for HPV.
		• The most common HPV types detected were a single infection with HPV 16 (62/121; 51%), then HPV 18 (34/121; 28%) followed by infections with multiple HPV types (8/121; 7%), HPV 56 and 58 (4/121; 3.3% each), HPV 31 and 42 (2/121; 1.7% each), and HPV 33, 51, 6, 66, and 68 (1/121; 0.8% each).
**Kuwait**		
Kapila et al.^29^	June 2017–May 2018	• **Population: **Cases initially diagnosed as ASCUS.
		• **Sample size: **180
		• The prevalence of high-risk HPV was 27/180 (15%).
		• High-risk HPV was present in 20/105 (19%) ASCUS cases, 1/58 (1.7%) cases with normal cytology, and 6/17 (35.3%) cases with endocervical abnormalities.
		• HPV 16 was positive in 5/27 (18.5%).
		• HPV 18/45 was positive in 1/27 (3.7%).
		• HPV 16 and 18/45 were positive in 8/27 (29.6%).
		• 13/27 (48.1%) were negative for both HPV 16 and 18/45, indicating the presence of other high-risk HPV genotypes (not genotypes 16 and 18/45).
Al-Awadhi et al.^30^	Nov 2016–May 2017	• **Population: **Immunocompetent men and women with anogenital warts who were scheduled for cryotherapy or laser treatment.
		• **Sample size: **156 total (129 men and 27 women).
		• The prevalence of high-risk HPV genotypes (HPV 16, 18, 33, and 38) was 54/156 (34.62%).
		• The prevalence of low-risk HPV genotypes (HPV 6 and 81) was 22/156 (14.1%)
		• The prevalence of common wart viruses (HPV 1a, 2, 7, 27b, 27, 57b, 57c, and 65) was 79/156 (50.6%).
		• HPV infection with a single type, two types, and three types was found in 88.4%, 9%, and 2.6% of patients, respectively.
		• A single HPV infection was found for the following genotypes: HPV 16 (44/156, 28.2%), HPV 27b (23/156, 14.7%), HPV 57c (22/156, 14.1%), HPV 6 (16/156, 10.3%), HPV 2 (15/156, 9.6%), HPV 65 (7/156, 4.5%), HPV 1a, 18, and 57b (2/156, 1.3% each), and HPV 7, 9, 27, 38, and 81 (1/156, 0.64% each).
		• Other genotypes found in double and triple infections additionally include HPV 4 and 33.
Mallik et al.^31^	Jul 2015–Sept 2017	• **Population: **Women diagnosed with epithelial abnormalities.
		• **Sample size: **749
		• High-risk HPV was positive in 229/749 (30.57%) of the cases with epithelial abnormalities; however, only 116 were genotyped.
		• 73/116 (62.93%) were negative for HPV 16, 18, and 45; 36/116 (31.06%) were positive for HPV 16; and 7/116 (6.03%) were positive for HPV 18/45.
AlRoomy et al.^32^	N/A	• **Population: **cervical samples with abnormal cytology were genotyped.
		• **Sample size: **330 total (282 samples with abnormal cytology and unknown HPV results, and 48 samples with normal cytology and known HPV results).
		• 153/282 (54.3%) samples with abnormal cytology were positive for HPV.
		• High-risk HPV types were detected in 115/190 (61%) samples with abnormal cytology.
		• Low-risk HPV types were detected in 69/190 (36%) samples with abnormal cytology.
		• Intermediate-risk HPV types were detected in only 6/190 (3%) samples with abnormal cytology.
		• Out of the high-risk HPV samples, HPV 16 was the most prevalent (39/115; 34%), followed by HPV 66 (15/115; 13%), HPV 53 (11/115, 9.6%), HPV 33 and 56 (10/115, 8.7%, each), HPV 18 (7/115, 6.1%), HPV 31 and 35 (6/115, 5.2% each), HPV 58 (3/115, 2.6%), HPV 39, 45, and 68 (2/115, 1.7% each), and HPV 59 and 73 (1/115, 0.9%).
		• Out of the intermediate-risk HPV samples, HPV 67 and 70 were the most common (2/6, 33% each), followed by HPV 84 and 87 (1/6, 16.7% each).
		• Out of the low-risk HPV genotypes identified, HPV 11 was the most common (25/69, 36.2%), followed by HPV 6 (21/69, 30.4%), HPV 90 (8/69, 11.6%), HPV 81 (7/69, 10.1%), HPV 61, 83, and 102 (2/69, 2.9% each), and HPV 54 and 106 (1/69, 1.4% each).
**Qatar**		
Elmi et al.^33^	Mar 2013–Aug 2014	• **Population: **Women of Arabic origin residing in Qatar.
		• **Sample size: **406
		• 33/406 (8.1%) tested positive for HPV.
		• 22/225 (9.8%) of Qatari women tested positive for HPV.
		• 11/181 (6.1%) of non-Qatari Arab women tested positive for HPV.
		• Among women with normal cytology, HPV was positive in 29/382 (7.6%).
		• Among women with abnormal cytology, HPV was positive in 4/24 (16.7%).
		• The most common high-risk genotype among women with normal cytology was HPV 35 (2/29, 6.9%), followed by HPV 33, 39, and 59 (1/29, 3.4% each).
		• The most common high-risk genotypes among women with abnormal cytology were HPV 16 and 59 (1/4, 25% each).
		• The most common low-risk genotypes among women with normal cytology were HPV 81 (10/29, 34.5%) and HPV 11 (9/29, 31%).
		• The most common low-risk genotypes among women with abnormal cytology were HPV 11, 81, and 90 (1/4, 25%).
**Oman**		
Al-Lawati et al.^34^	Sept 2014–Apr 2015	• **Population: **Married Omani women, 18–68 years, attending the gynecology clinic.
		• **Sample size: **258
		• HPV was positive in 46/258 (17.8%).
		• The prevalence of HPV among women with normal cytology was 39/231 (16.9%).
		• The prevalence of HPV among women with abnormal cytology was 6/16 (37.5%).
		• Out of the 46 HPV positive cases, there were 65 positive results encompassing 22 different genotypes (due to 11 cases testing positive for more than 1 HPV type).
		• The most common high-risk genotypes included HPV 82 (7/65, 10.77%) followed by HPV 68 (6/65, 7.69%), HPV 18, 53, and 56 (4/65, 6.15% each), HPV 51, 58, and 73 (3/65, 4.62% each), HPV 39 (2/65, 3.08%), and HPV 16, 31, 33, 35, 52, and 66 (1/65, 1.54%).
		• The most common low-risk genotypes included HPV 54 (8/65, 12.31%), followed by HPV 42 (5/65, 7.69%), HPV 44 (4/65, 6.15%), HPV 6 (3/65, 4.62%), HPV 43 (2/65, 3.08%), and finally HPV 11 and HPV 70 (1/65, 1.54% each).
**Bahrain**		
Alnoaimi et al.^36^	Jan 2017–Apr 2023	• **Population: **HPV positive cases with both normal and abnormal Pap smear findings.
		• **Sample size: **100
		• 24/100 (24%) had HPV 16 (16/24, 66.7% had an abnormal Pap smear).
		• 6/100 (6%) had HPV 18/45 (4/6, 66.7% had an abnormal Pap smear).
		• 62/100 (62%) had other HPV genotypes (7/62, 11.3% had an abnormal Pap smear)
		• 5/100 (5%) had HPV 16 and other high-risk HPV (non-16/18/45) coinfection.
		• 2/100 (2%) had HPV 18/45 and other high-risk HPV (non-16/18/45) coinfection.
		• 1/100 (1%) had HPV 16 and HPV 18/45 coinfection.
**Qatar, Bahrain, KSA, and UAE**		
Ali et al.^19^	N/A	• **Population: **Women attending the outpatient clinic.
		• **Sample size:** 2,478 total (1276 from KSA, 728 from Qatar, 409 from UAE, and 65 from Bahrain)
		• 520/2,478 (21%) tested positive for high-risk HPV.
		• High-risk HPV prevalence was highest among women in Qatar (228/728; 31.3%), followed by women residing in Bahrain (13/65; 20%), KSA (219/1276; 17.2%), and the UAE (60/409; 14.7%).
		• HPV positivity rate was higher among women with abnormal cytology (219/433, 50.6%) compared to women with normal cytology (301/2045, 14.7%).
		• 90/520 (17.3%) tested positive for HPV 16.
		• 14/520 (2.7%) tested positive for HPV 18.
		• 5/520 (1%) tested positive for HPV 16/18 coinfection.
		• Prevalence of HPV 16 with other high-risk HPV coinfection was 62/520 (11.9%).
		• Prevalence of HPV 18 with other high-risk HPV coinfection was 6/520 (1.2%).
		• Prevalence of coinfection with HPV 16, 18 and other high-risk HPV genotypes was 12/520 (2.3%).
		• Prevalence of other high-risk HPV was 331/520 (63.7%).

ASCUS, atypical squamous cells of undetermined significance; HSIL, high-grade squamous intraepithelial lesion; KSA, Kingdom of Saudi Arabia; UAE, United Arab Emirates.
